# Influence of deep learning image reconstruction and adaptive statistical iterative reconstruction-V on automated Alberta stroke program early CT score- evaluation

**DOI:** 10.1007/s00234-026-03938-5

**Published:** 2026-02-18

**Authors:** Estelle Akl, Daniel Cantré, Matthias Lütgens, Wiebke Hermann, Sönke Langner, Marc-André Weber, Ann-Christin Klemenz, Felix G. Meinel, Ebba Beller

**Affiliations:** 1 Institute and Policlinic of Radiology, Pediatric Radiology and Neuroradiology, University Medical Center Rostock, Rostock, Germany; 2 Department of Neurology, University of Rostock, Rostock, Germany

**Keywords:** Stroke, ASPECTS, Computed tomography, Deep learning, Iterative reconstruction

## Abstract

**Purpose:**

The Alberta Stroke Program Early CT Score (ASPECTS) and advances in CT reconstruction play important roles in the neurodiagnostic workflow. This study examines the effect of these reconstruction techniques on automated ASPECTS.

**Methods:**

In this retrospective study, 173 patients (median age 79 years, 39% female) with suspected middle cerebral artery infarction underwent non-contrast CT scans reconstructed with Filtered Back Projection (FBP), ASIR-V (30% and 60%), and DLIR (low, medium, and high). Automated ASPECTS were analyzed, with FBP as the reference standard.

**Results:**

Bland–Altman analysis revealed a mean bias with ASIR and DLIR underestimating ASPECTS compared to FBP. This underestimation was less pronounced for ASIR-V 30% (-0.057 ) and DLIR-L (-0.069) than for ASIR-V 60% (-0.126), DLIR-M (-0.121), and DLIR-H (-0.086). The region with the greatest overestimation relative to FBP was M3 (*n* = 23), while the region with the greatest underestimation was the insular ribbon (*n* = 51). Regarding the ASPECTS < 6 threshold, most patients were reclassified from ASPECTS ≤ 5 to ASPECTS ≥ 6 with DLIR-M (*n* = 5), which also showed the strongest agreement with expert consensus (κ = 0.352).

**Conclusion:**

Both ASIR-V and DLIR resulted in only minor underestimation of ASPECTS compared to FBP. However, most patients became eligible for endovascular therapy due to ASPECTS reclassification with DLIR-M. DLIR-M also exhibited the highest agreement with expert consensus for automated ASPECTS. Therefore, careful selection of reconstruction parameters, as well as further optimization and standardization of these techniques, is essential for broader application in stroke imaging.

**Supplementary Information:**

The online version contains supplementary material available at 10.1007/s00234-026-03938-5.

## Highlights

- CT Reconstruction techniques influence automated ASPECT scoring reliability.
- Insula, as well as the cortical M3 ASPECTS region, seemed more susceptible to algorithmic variability.
- More patients were overestimated from ASPECTS ≤ 5 to ASPECTS ≥ 6, making them available for endovascular therapy.
- DLIR-M demonstrated the highest agreement between the automated ASPECTS assessment and the expert-derived ground truth.

**Supplementary Information:**

The online version contains supplementary material available at 10.1007/s00234-026-03938-5.

## Background

The Alberta Stroke Program Early Computed Tomography Score (ASPECTS) is widely used to evaluate early ischemic changes in patients with acute ischemic stroke, providing a standardized approach to estimate the extent of the ischemic region [1]. In the actual guidelines, an ASPECTS score of 6 or higher is a requirement or criterion for selecting patients who should receive endovascular therapy [2, 3]. ASPECTS is also used for the evaluation of stroke severity and prognosis in acute ischemic stroke [4, 5]. However, recent studies support endovascular thrombectomy (EVT) as safe and effective in selected patients with large ischemic cores and low ASPECTS, particularly in early (≤ 6-hour) time windows, even though overall outcomes remain heterogeneous [6, 7]. Some studies even suggest that very low ASPECTS (0–2) may still be associated with functional benefit from EVT in otherwise eligible patients, with factors such as the extent of tissue damage, infarct topography, disruption of structural brain network connectivity, and selective neuronal loss likely playing a more significant role in determining individual prognosis than infarct volume alone [8]. Another limitation of ASPECT scoring, which is traditionally conducted manually, is the considerable variability among observers. To address these limitations, automated ASPECTS tools based on artificial intelligence (AI), including deep learning (DL) and convolutional neural networks, have emerged, capable of complex tasks such as feature extraction, classification, and segmentation in medical imaging [9, 10].

The integration of AI into stroke imaging has also transformed other aspects of the diagnostic workflow [11]. Historically, Filtered Back Projection (FBP) was the primary method for CT image reconstruction due to its computational efficiency and numeric stability. Although FBP relies on a relatively simple reconstruction process, it performs adequately in most clinical situations. However, FBP has well-documented limitations, including its inability to effectively manage noise, particularly at lower radiation doses or with obese patients [12]. Iterative Reconstruction (IR) was subsequently introduced as an alternative post-processing method for CT reconstruction, using different mathematical algorithms to improve noise management and reduce dose. Adaptive statistical IR (ASIR; GE Healthcare, Waukesha, WI) is a new generation of such post-acquisition image-processing technique, correcting image data by changing the photon statistics in X-ray attenuation and reducing noise iteratively in the image domain [13]. More recently, Deep Learning Image Reconstruction (DLIR) has emerged as a powerful alternative to FBP and IR, enhancing noise reduction and improving image quality, while maintaining an acceptable radiation dose. DLIR utilizes trained artificial neural networks to refine images and improve diagnostic confidence, particularly in challenging imaging conditions. Recent studies have demonstrated that DLIR-based methods outperform traditional IR by producing images with reduced noise and superior contrast-to-noise ratios, thereby enhancing the diagnostic potential for various pathologies [12, 14, 15].

In this study, we aim to evaluate the impact of different CT reconstruction algorithms on the performance of automated ASPECT scoring in patients with acute ischemic stroke. Understanding the differences among these reconstruction algorithms could help optimize imaging protocols and ultimately improve clinical outcomes for stroke patients.

## Methods

### Patient selection and study design

This study was designed as a retrospective, single-center study. Patients were identified by a retrospective search of our radiology information system (Centricity 5.0, GE Healthcare, Barrington, Illinois). Inclusion criteria were patients who received a non-contrast head CT (NCCT) and, optionally, CT angiography (CTA) or CT perfusion at our institution for suspicion of an acute stroke in the middle cerebral artery (MCA) territory between May 13, 2024, and November 19, 2024 (*n* = 490). We excluded patients with other pathologies (e.g. tumor, trauma, headache…), (*n* = 159), symptoms suggesting an involvement of the anterior cerebral artery territory and posterior circulation (*n* = 64), hemorrhagic strokes (*n* = 39), large, chronic, ischemic areas in the MCA territory (*n* = 15) as well as acute and subacute strokes with more than 24 h after the onset of MCA stroke symptoms (*n* = 8). Twenty patients were excluded due to technical issues with the automated ASPECTS software, and 12 due to incomplete datasets. One patient was excluded due to artifacts caused by a cochlear implant (Suppl. Figure 1). Of the remaining 173 patients, baseline NIHSS recordings were found for 150 after reviewing electronic patient charts.

### Human ethics and consent to participate declarations

The study protocol was approved by the Ethics Committee of Rostock University Medical Faculty (A 2024 − 0178, date of approval: November 11, 2024), and informed consent to participate was waived due to the retrospective nature of this study. The study was conducted in compliance with the Declaration of Helsinki in its current form.

### CT acquisition protocol

All included patients underwent CT imaging as per the standard acute stroke protocol. The radiologist on duty was informed of the stroke patient by the neurologist via a “code stroke” alarm call. Patients with symptoms lasting 6 h or less and suspected stroke in the anterior vascular circulation underwent NCCT, CT perfusion, and CTA of the supra-aortic and intracranial arteries. Patients with symptom onset between 6 and 24 h and also suspected stroke in the anterior vascular circulation underwent NCCT and CTA. CT perfusion was then only performed if a large vessel occlusion was detected. Patients with an unknown time window or wake-up stroke, as well as patients with suspected involvement of the posterior circulation, underwent cMRT and were not included in this study. NCCT, CT perfusion, and CTA were conducted using a 128-slice CT scanner (Revolution CT, GE Healthcare). Scanning parameters for NCCT included a slice thickness of 0,625 mm, a reconstruction of 3,0 mm, a voltage of 120 KV, and a tube current modulation, determined based on the Scout in mA. The median CT dose index was 47 mGy (IQR 46–52 mGy).

### Image reconstruction and analysis

**Fig. 1:**
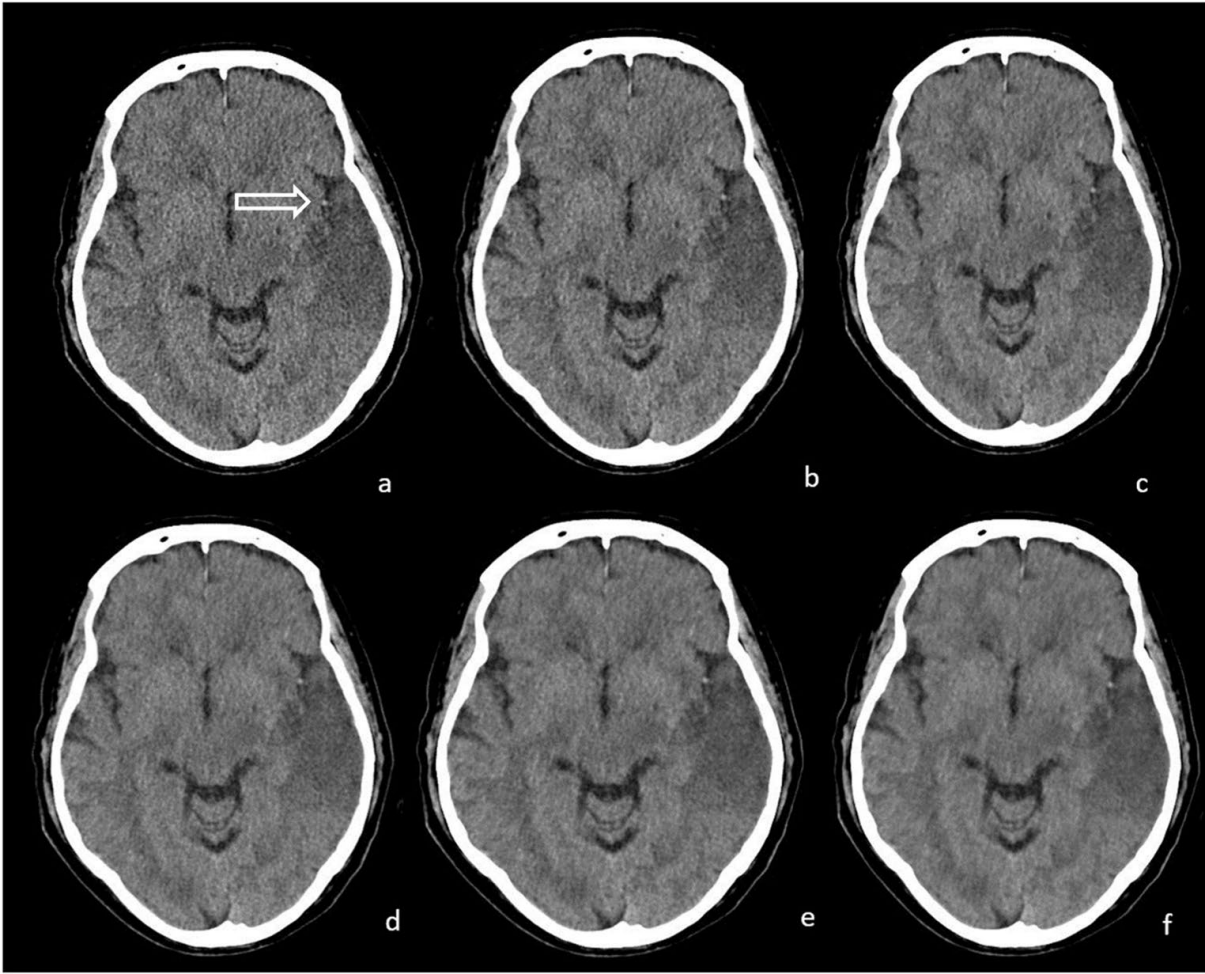
Early ischemic changes visualized by different CT reconstruction techniques. Axial non-contrast CT images demonstrating early ischemic changes in the left temporal region with a hyperdense middle cerebral artery sign (white arrow in **a**). Images were reconstructed using six different algorithms: Filtered Back Projection (FBP) (**a**), Adaptive Statistical Iterative Reconstruction-V (ASIR-V) at 30% (**b**) and 60% (**c**), and Deep Learning Image Reconstruction (DLIR) at low (L) (**d**), medium (M) (**e**), and high (H) (**f**) strengths

For all patients, NCCT images of the brain were reconstructed on the Revolution CT scanner (software version 2.1B) using standard Filtered Back Projection (FBP), ASIR-V at 30% and 60% strength, as well as three strength levels of DLIR (low (DLIR_L), medium (DLIR_M), and high (DLIR_H); “TrueFidelityTM”, GE Healthcare, Chicago) (Fig. 1). ASIR-V offers the possibility to blend IR with FBP images, ranging from 0% ASIR-V (equals 100% FBP) to 100% ASIR-V (equals 0% FBP) [13]. ASIR-V at 30% strength aligns with the vendor’s recommendation for a general-use range of 30–50% ASIR [16]. ASI16R-V at 60% strength was also chosen for this study to investigate a greater influence of IR over FBP. DLIR can be selected with low, medium, or high reconstruction strengths according to different dose and noise reduction requirements [17]. However, it has not yet been established whether and to what extent DLIR provides advantages when applied to automated ASPECTS.

The image slice thickness used for automated ASPECT score determination was reconstructed using the standard convolution kernel and a slice thickness of 2.5 mm, as recommended by the manufacturer for optimal ASPECT score evaluation. ASPECTS was obtained automatically using commercially available StrokeSENS ASPECTS software (AW server 3.2 software, GE HealthCare). The software uses a Convolutional Neural Network (CNN) that was trained to detect and segment tissue with early ischemic changes by learning global and local features of healthy and ischemic tissue. Since it is a CNN-based prediction, there is no direct comparison of Hounsfield units between contralateral and affected regions. A CT template is co-registered to the input CT image via an iterative registration algorithm, allowing for the ASPECT regions to be localized and delineated in the original input CT volume. Early ischemic changes and ASPECTS regions are synthesized to determine regions that are affected, based on an overlap ratio between early ischemic changes and ASPECT regions of > = 15%. The ten specific ASPECTS regions within the middle cerebral artery (MCA) territory include the caudate nucleus (C), lentiform nucleus (L), internal capsule (IC), insular ribbon (I), as well as the cortical regions M1, M2, and M3 (anterior, middle, and posterior third of the lower MCA territory, respectively), and M4, M5, and M6 (anterior, middle, and posterior third of the higher MCA territory, respectively). The presence of a hyperdense artery sign is not required for segmentation. Patient age or time of symptom onset are not integrated into the software algorithm, and no semi-automated option is available to correct the automated segmentation. However, the suspected side of ischemia can be manually selected by the user. Each region demonstrating signs of early ischemic changes is deducted from the total of ten, resulting in a final ASPECTS ranging from 0 (all areas affected by early ischemic changes) to 10 (no changes) [10]. ASPECTS ranging from 0 to 4 were additionally defined as low, from 5 to 7 as intermediate, and from 8 to 10 as high.

Ground truth was established by expert consensus between one radiology and one neuroradiology consultant (with 11 and 12 years of experience in stroke imaging) by using all available imaging at the acute stage, including NCCT with FBP, ASIR, and DLIR technique, CT angiography, CT perfusion, Digital Subtraction Angiography, and follow-up CT or MRI. Follow-up NCCT was routinely performed 24 h after stroke thrombolysis and/or thrombectomy. In patients with suspected ischemic stroke whose initial CT scan was inconclusive—for example, when the ASPECTS or perfusion findings did not adequately account for the acute neurological deficit—a follow-up MRI was typically performed the following day. Both readers were blinded to the automated ASPECTS rating results.

### Statistical analysis

Statistical analysis was performed using GraphPad Prism version 9.0.0 (GraphPad Software LLC). FBP was selected as the reference for comparison with ASIR and DLIR. Bland-Altman plots were used to determine the mean difference and limits of agreement between FBP and the other reconstruction techniques [18–20]. The agreement between inconsistent automated ASPECTS ratings of FBP, ASIR, and DLIR reconstructions and the ground truth was assessed using weighted kappa analysis. P-values ≤ 0.05 were considered significant.

## Results

### Patient characteristics

The final study population included 173 participants with suspected MCA infarction and symptom onset within 24 hours. The median time from stroke onset to CT imaging was 3 hours (IQR 1.5-6 hours), and the median National Institutes of Health Stroke Scale score was 4 (IQR 1–10). They had a median age of 79 years (IQR 71–86 years), and 39% (n = 68) were female. In 117 (68%) patients, the automated ASPECTS results did not differ across different CT reconstruction techniques. In 56 patients (32%), one or more score results of the automated ASPECTS differed between the different CT reconstruction techniques. 

### Agreement of total ASPECTS between FBP and ASIR-V or DLIR

**Fig. 2:**
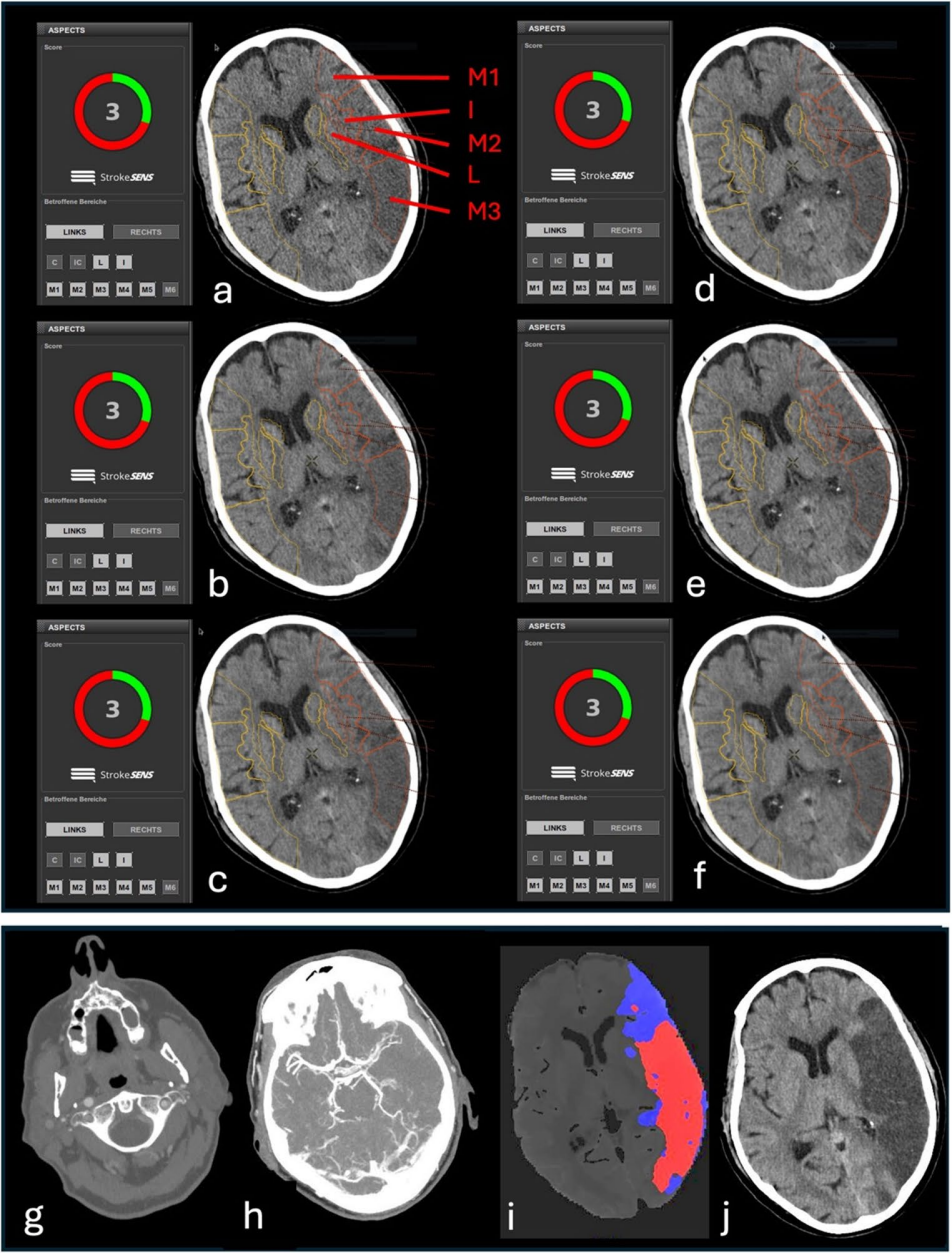
Consistent, automated ASPECTS results of a patient with MCA infarction. Automated ASPECTS analysis of a NCCT of a 69-year-old male patient with a baseline NIHSS of 21, as well as stroke symptoms two hours prior to imaging, which included eye deviation to the right, right-sided paresis, and global aphasia. ASPECTS was calculated for each different reconstruction technique: Filtered Back Projection (FBP) (**a**); Adaptive Statistical Iterative Reconstruction-V (ASIR-V) at 30% (**b**) and 60% (**c**), and Deep Learning Image Reconstruction (DLIR) at low (L) (**d**), medium (M) (**e**), and high (H) (**f**) strength. Areas with presumed early ischemic changes were automatically contoured in red (I, Insula; L, lentiform nucleus; M1-M3), and automated ASPECTS were displayed on the left with a score of 3 for each CT reconstruction technique. CTA imaging with occlusion of the left internal carotid artery (black arrow, **g**) and left distal M1 segment (black arrow, **h**). A large infarct core volume of 91 ml compared to a penumbra volume of 115 ml was estimated by CT perfusion (**i**), making the patient ineligible for thrombectomy. Follow-up imaging after 24 h with extensive infarction of the MCA territory (**j**)

Examples of ASPECTS analysis of different CT reconstruction techniques in a representative patient are shown in Fig. 2. Bland-Altman plots of all reconstruction techniques (ASIR-V at 30% and 60%, DLIR-L, DLIR-M, and DLIR-H) compared to FBP as the reference standard were obtained. ASIR-V 30% showed the best agreement regarding ASPECTS compared to FBP, with a mean bias of − 0.057, whereas ASIR-V 60% had the highest deviation in scoring results, with a mean bias of − 0.126. The mean bias for DLIR-L, DLIR-M, and DLIR-H was – 0.069, − 0.121, and – 0.086 points, respectively. Detailed results of the Bland–Altman analysis are shown in Table 1.

**Table 1:** Bland–altman analysis for ASPECTS of FBP vs. ASIR-V and DLIR

Comparison			95% Limits of agreement	
	Mean Bias	SD of Bias	Upper	Lower
FBP	Reference			
ASIR-V 30%	-0.057	0.489	-1.017	0.902
ASIR-V 60%	-0.126	0.605	-1.311	1.058
DLIR-L	-0.069	0.632	-1.308	1.170
DLIR-M	-0.121	0.682	-1.457	1.216
DLIR-H	-0.086	0.767	-1.589	1.417

*FBP* serves as the reference standard. *ASIR-V* adaptive statistical iterative reconstruction-V, *DLIR* deep learning-based image reconstruction in low (L), medium (M), or high (H) strength, *FBP* filtered back projection

### Impact on the classification of patients into risk categories based on ASPECTS

**Fig. 3:**
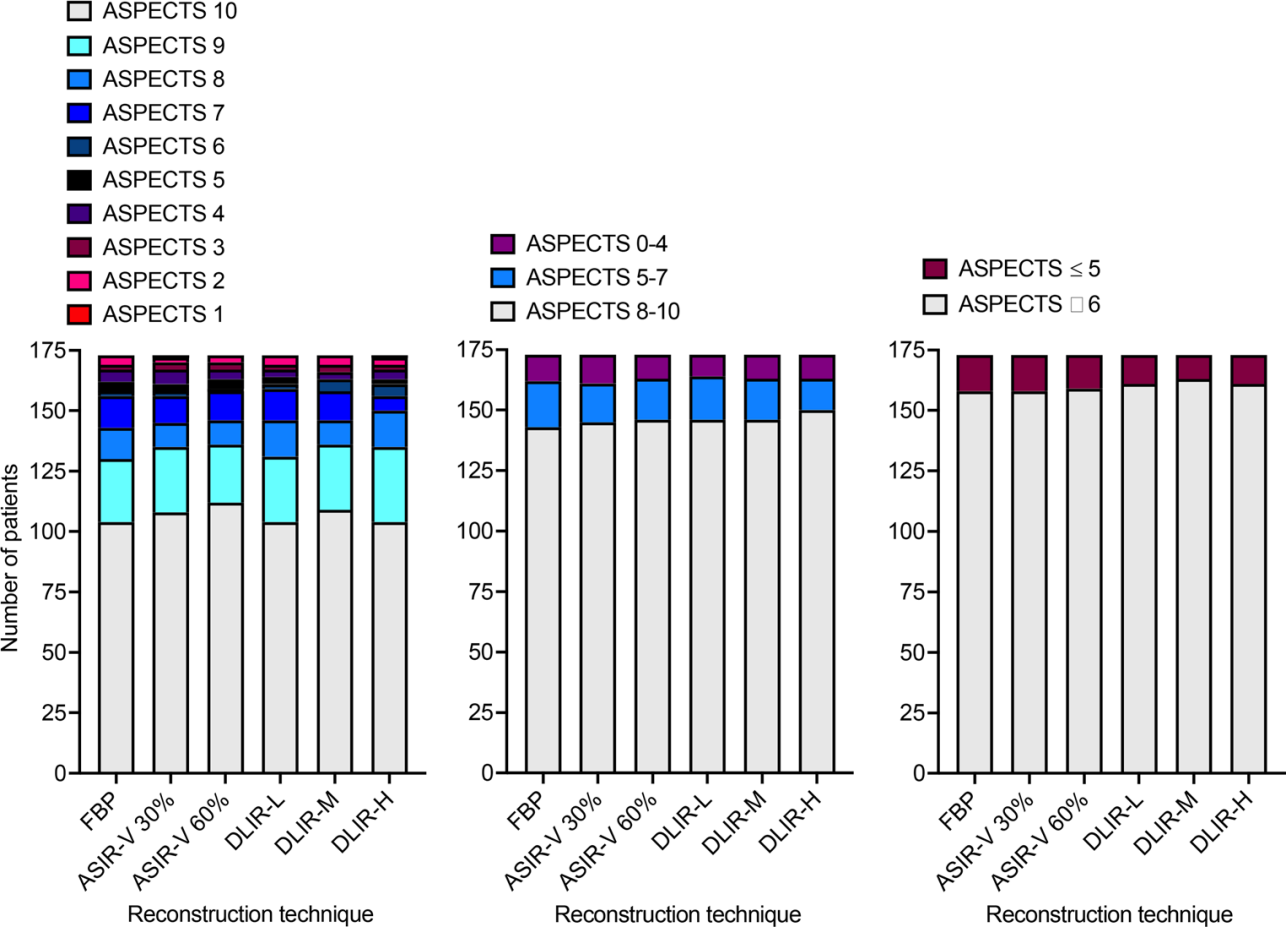
Distribution of ASPECTS categories by reconstruction technique: ASIR-V, adaptive statistical iterative reconstruction-V; FBP, filtered back projection; DLIR, deep learning- based image reconstruction in low (L), medium (M), or high (H) strength

Reclassifications of ASPECTS categories using FBP as the reference standard were observed for each reconstruction technique; see Fig. 3; Table 2 for details. Most of the re-classifications were from intermediate (score 5–7) to high ASPECTS (score 8–10), with three and five patients, using ASIR-V at 30% and 60% strength, as well as seven, five, and ten patients, using DLIR at low, medium, and high strength. However, patients were also re-classified from a high to intermediate ASPECTS, e.g. two patients regarding ASIR-V at 60% strength, as well as four, two, and three patients regarding DLIR at low, medium, and high strength, respectively. With only two patients, most reclassifications were from low to intermediate ASPECTS with DLIR at low strength. Three, five, and four patients were re-classified from ASPECTS ≤ 5 to ASPECTS ≥ 6 with DLIR at low, medium, and high strength.

**Table 2:** Change in ASPECTS categories comparing FBP to ASIR-V and DLIR

ASPECTS category	Net change in number of patients in risk groups									
	ASIR-V 30%		ASIR-V 60%		DLIR-L		DLIR-M		DLIR-H	
high (8–10)	-1	+ 3	-2	+ 5	-4	+ 7	-2	+ 5	-3	+ 10
moderate (5–7)	-4	+ 1	-5	+ 3	-7	+ 6	-5	+ 3	-10	+ 4
low (0–4)	0	+ 1	-1	0	-2	0	-1	0	-1	0
≥ 6	-1	+ 1	0	+ 1	0	+ 3	0	+ 5	-1	+ 4
≤ 5	-1	+ 1	-1	0	-3	0	-5	0	-4	+ 1

*ASIR-V* adaptive statistical iterative reconstruction-V, *DLIR* deep learning-based image reconstruction in low (L), medium (M), or high (H) strength, *FBP* filtered back projection. FBP served as the reference standard

### Impact on regional distribution of ischemic changes based on ASPECTS

**Fig. 4:**
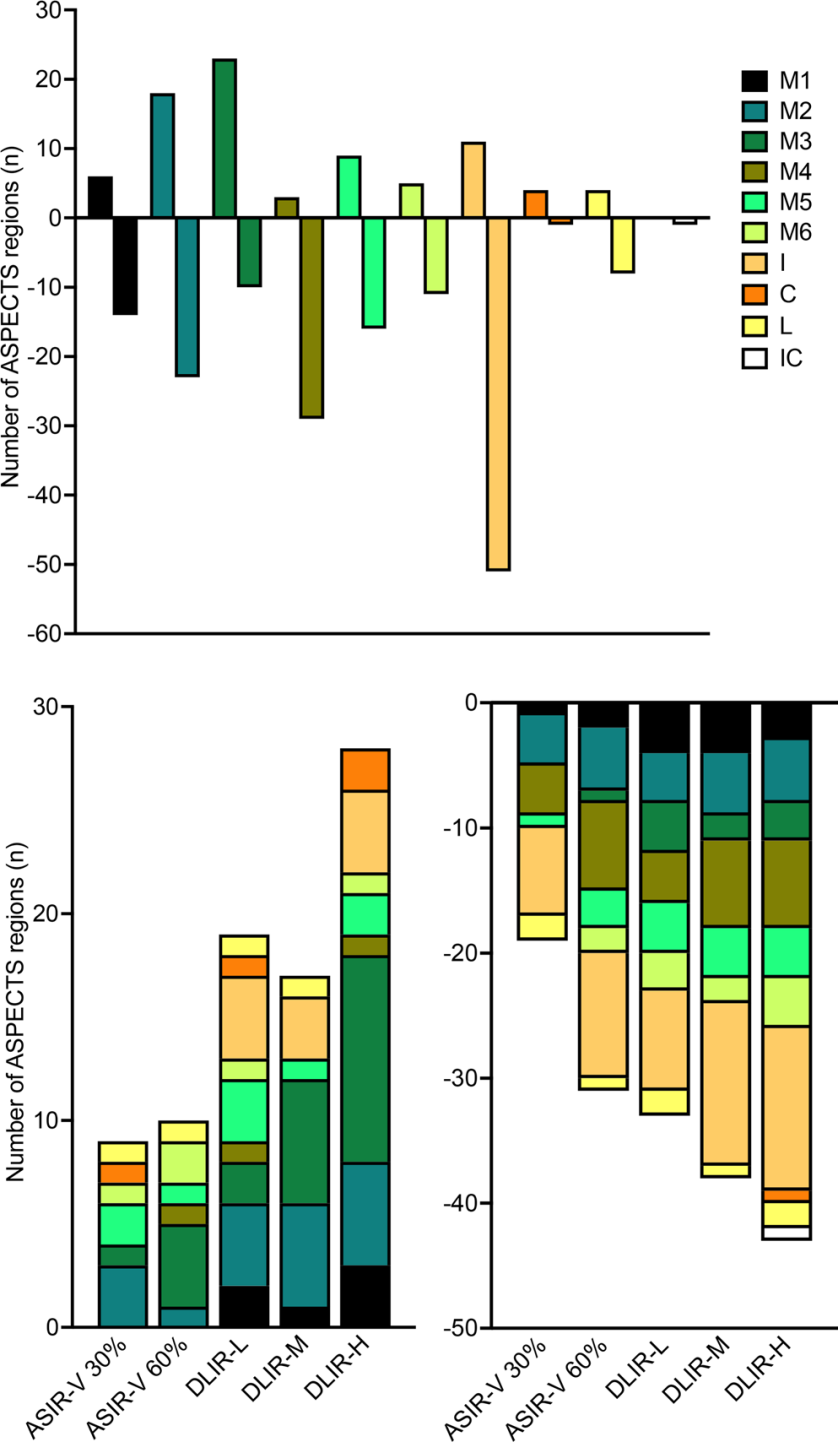
Over- and underestimation of ischemic change per ASPECTS region as the sum of all reconstruction techniques in comparison to FBP (upper row) and of each reconstruction technique compared to FBP (lower row). ASIR-V, adaptive statistical iterative reconstruction-V; FBP, filtered back projection; DLIR, deep learning-based image reconstruction in low (L), medium (M), or high (H) strength; C, caudate nucleus; L, lentiform nucleus; IC, internal capsule; I, insular ribbon; M1-6, six cortical areas of the middle cerebral artery territory

**Fig. 5:**
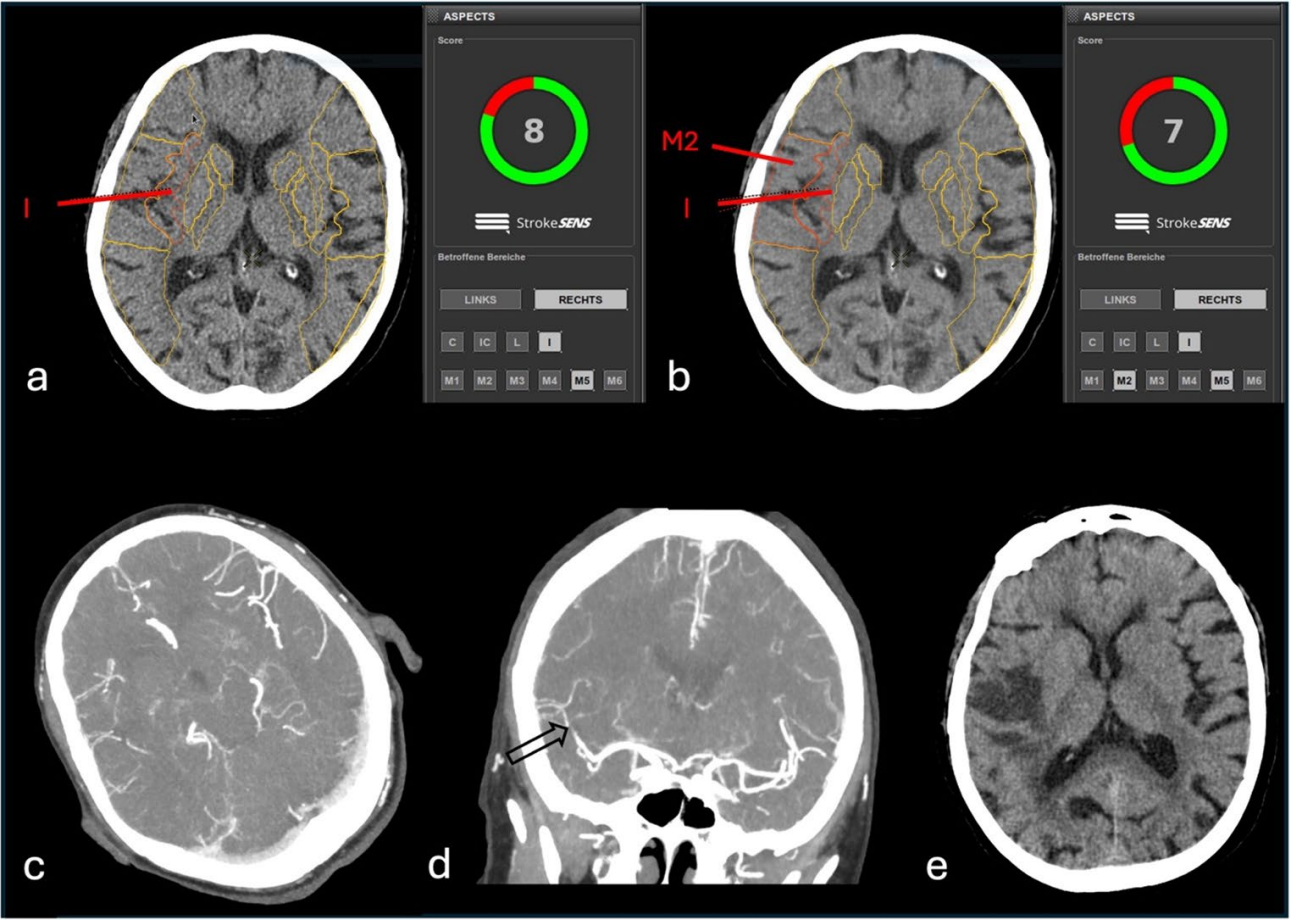
Overestimation of automated ASPECTS comparing FBP with DLIR-H. Automated ASPECTS analysis of a NCCT of a 72-year-old female patient with a baseline NIHSS of 12, as well as stroke symptoms 12 h prior to imaging, which included dysarthria, left arm paralysis, and neglect. With the application of FBP (**a**), early ischemic changes were identified of the insula and M5 region of the right hemisphere, resulting in an ASPECTS of 8, whereas with the application of DLIR-H (**b**), the M2 cortex area of the right hemisphere was additionally marked as an early ischemic region (in red), resulting in an ASPECTS of 7. CTA imaging reformatted as 10-mm-thick axial (**c**) and coronal MIP (**d**) with occlusion of the right M2 segment (black arrow). Mechanical thrombectomy was not performed due to the time window of 12 h and the thrombus location. Follow-up imaging after 48 h with partial infarction of the MCA territory involving the M2 region (**e**)

**Fig. 6:**
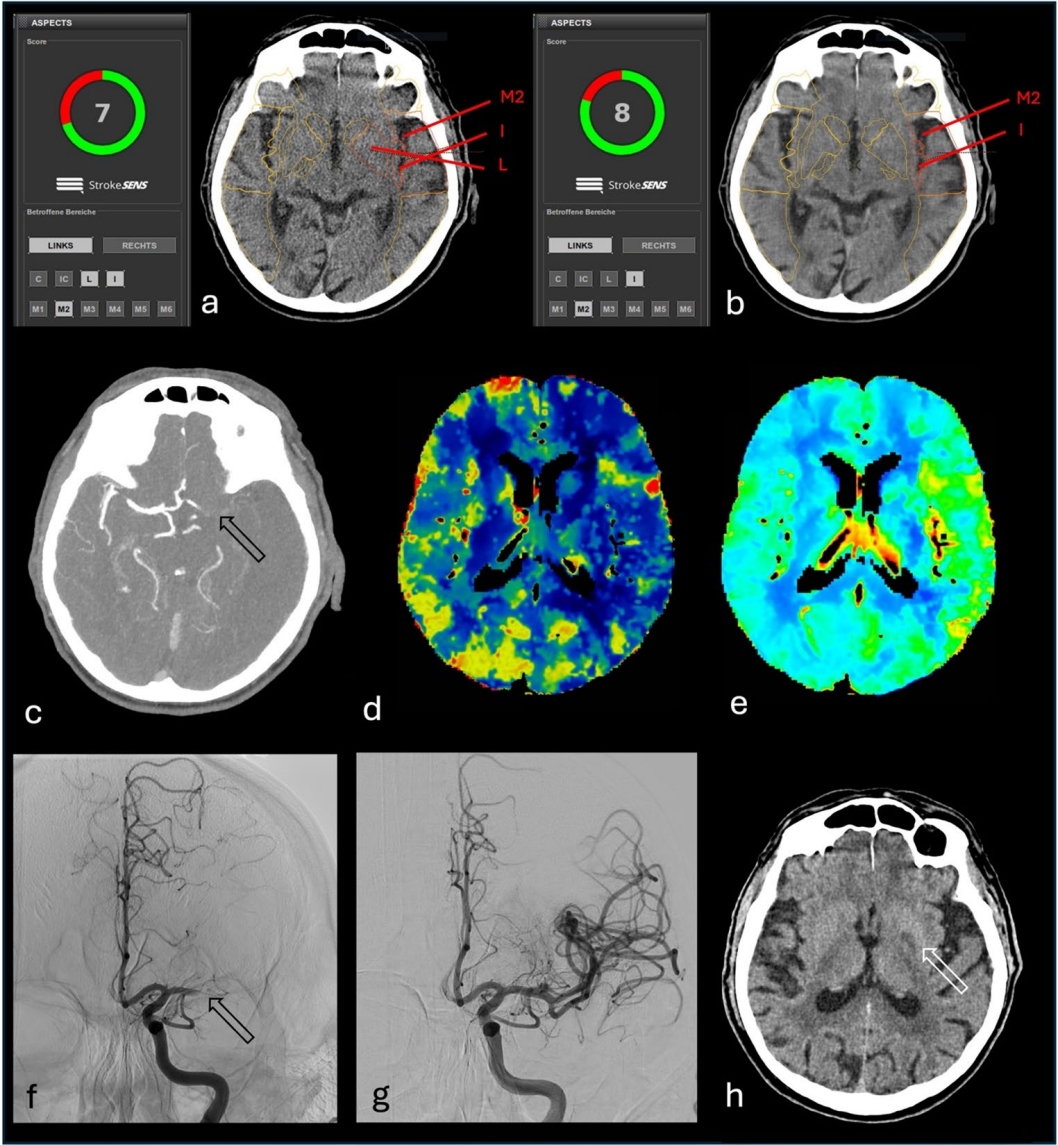
Underestimation of automated ASPECTS comparing FBP with DLIR-H. Automated ASPECTS of a NCCT of a 89-year-old male patient with a baseline NIHSS of 23, as well as right-sided hemiparesis with onset 1 h prior to imaging. With FBP (**a**), early ischemic changes were identified in the insula (I), lentiform nucleus (L), as well as in the M2-region of the left middle cerebral artery territory (in red), resulting in an automated ASPECTS of 7, whereas with DLIR-H (**b**), only the insula and M2-region was marked as affected, resulting in an ASPECTS of 8. CTA imaging reformatted as 10-mm-thick axial MIP (**c**) showed a gradual contrast decline located at the level of the carotid T (black arrow). CT perfusion revealed decreased cerebral blood flow (**d**) and increased cerebral blood volume (**e**) of the left MCA territory. Digital subtraction angiography showed acute left M1 proximal occlusion (black arrow, **f**), and mechanical thrombectomy was performed with a CT-to-reperfusion delay of 64 min (**g**). Follow-up imaging after 24 h showed only a small infarcted area in the left globus pallidus (white arrow, **h**)

Over- and underestimation of ischemic change per ASPECTS region of all reconstruction techniques (ASIR-V at 30% and 60%, DLIR-L, DLIR-M, and DLIR-H) compared to FBP as the reference standard is shown in Fig. 4. The region with the highest overestimation, summarized from all reconstruction techniques in comparison to FBP was M3 (*n* = 23), followed by M2 (*n* = 18), insula (*n* = 11), M5 (*n* = 9), M1 (*n* = 6), M6 (*n* = 5), lentiform nucleus as well as caudate nucleus (*n* = 3) and M4 (*n* = 3), (Fig. 5). No overestimation was found for the internal capsule (*n* = 0). The region with the highest underestimation compared to FBP was the insular ribbon (*n* = 51) (Fig. 6), followed by M4 (*n* = 29), M2 (*n* = 23), M5 (*n* = 16), M1 (*n* = 14), M1 (*n* = 11), M3 (*n* = 10), lentiform nucleus (*n* = 8) followed by caudate nucleus as well as the internal capsule (*n* = 1). With regards to the reconstruction techniques, the highest number of overestimation of ASPECTS regions, compared to FBP, was found with DLIR-H (*n* = 28), followed by DLIR-L (*n* = 19), DLIR-M (*n* = 17), ASIR-60% (= *n* = 10), and ASIR-30% (*n* = 9). The highest number of underestimation of ASPECTS regions, compared to FBP, was found with DLIR-H (*n* = 43), followed by DLIR-M (*n* = 38), DLIR-L (*n* = 33), ASIR-60% (*n* = 31) and ASIR-30% (*n* = 19).

### Agreement between consensus and automated ASPECTS of FBP, ASIR, and DLIR

In 56 patients (32%), inconsistent results of the automated ASPECTS were found between different CT reconstruction techniques and were compared to expert consensus. DLIR-M showed the highest agreement with expert consensus (κ = 0.352), followed by ASIR-60% (κ = 0.346), ASIR-30% (κ = 0.291), DLIR-L (κ = 0.263), FBP (κ = 0.229), and DLIR-H (κ = 0.220).

## Discussion

In this study, we investigated the effects of CT reconstruction techniques on automated ASPECTS scores in patients with suspected MCA infarction. In total, ASIR-V and DLIR reconstructions showed only a minor underestimation of ASPECTS results compared to FBP. Regarding the ASPECTS < 6 threshold, however, more patients were overestimated when transitioning from ASPECTS ≤ 5 to ASPECTS ≥ 6, making them eligible for endovascular therapy, a phenomenon that was more pronounced for DLIR, especially DLIR-M, than for ASIR-V. Interestingly, the ASPECTS region with the highest overestimation compared to FBP was M3, and the region with the highest underestimation was the insular ribbon. The highest number of over- and underestimation of ASPECTS regions was found with DLIR-H. When comparing the inconsistent automated ASPECTS results across different CT reconstruction techniques and expert consensus as the ground truth, DLIR-M showed the highest agreement.

Over the last decade, iterative reconstruction (IR) algorithms have significantly advanced CT technology as a potential alternative to FBP [12, 15, 21, 22]. These methods improve image quality and reduce radiation dose, making them a criterion standard in CT imaging [14, 21–23]. Unlike IR and FBP, DLIR uses artificial neural networks to iteratively learn to eliminate noise while preserving anatomical details, thereby representing another step forward in both image quality and dose optimization [15, 22]. All advancements in CT reconstruction techniques have reshaped the landscape of neuroradiological CT imaging [24–27] and may therefore also hold significant potential to refine automated ASPECTS, a key tool for future AI-based assessment of acute ischemic stroke.

Similar studies have primarily focused on the agreement between ASPECTS scores obtained by automated software and those determined by human readers, showing overall good agreement [28, 29] or even improvements in the interobserver agreement and accuracy of neuroradiologists and neurologists in their interpretation of ASPECTS [30]. Löffler et al. compared ASPECTS assessments, which were based on hybrid IR or iterative model reconstruction (IMR) and carried out by humans and software in comparison to a consensus reference. They reported that the automated software improved IMR, which can further reduce noise levels and improve image contrast at a given dose level and slice thickness compared with hybrid IR [31]. Seker et al. NECT compared automated ASPECTS of FBP with Sinogram-Affirmed Iterative Reconstruction (SAFIRE) in strength levels 2 and 4 out of 5. This study included 43 patients with acute occlusion of the M1 segment of the middle cerebral artery and found that automated ASPECTS of SAFIRE at a strength level of 2 showed the highest consistency compared with a ground truth provided by an expert with unrestricted data access [32]. However, in both studies mentioned above, different reconstruction techniques were investigated in comparison to our study, and a detailed analysis of the ASPECTS categories and regions was not performed. Konno et al. found no significant differences in the ASPECTS evaluation of 30 patients when comparing different CT reconstruction techniques (FBP, ASIR-V at 50%, and DLIR), which were similar to our study. However, in this study only human readers were used for ASPECTS assessment and no automated software [33].

In our study, the highest number of over- and underestimation of ASPECTS regions was observed with DLIR-H. The insula was the most frequently underestimated ASPECTS region, while overestimation was most notable for the cortical M3 ASPECTS region, suggesting that both regions are more susceptible to algorithmic variability, e.g. due to difficulties in precisely defining anatomical borders. On the other hand, subcortical regions, such as the internal capsule, lentiform nucleus, and caudate nucleus, displayed greater consistency across reconstruction techniques, suggesting these regions are less influenced by algorithmic variability. Austein et al. also found disagreement in the characteristics of ASPECTS regions scored, which was most notable for the insula and internal capsule, when comparing between humans, and two different automated ASPECTS packages. They also reported variability in cortical scoring (M1–M6), which they concluded might be due to the challenges of consistently defining the anatomical borders of these regions [29]. Interestingly, van Horn et al., who evaluated interrater reliability in a large group of human readers, found that the ASPECTS region with the highest level of agreement was the insular cortex, and the lowest was the M3 region [34].

The most important clinical use of ASPECTS is to guide decisions on eligibility for thrombolysis and endovascular therapy [1, 18]. Variability in ASPECTS due to reconstruction technique, such as over- or underestimation of the total score, may significantly influence patient outcomes by altering the choice of therapy. According to established guidelines, an ASPECTS score of 6 or higher is a requirement or criterion for endovascular therapy [2, 3]. In our study, most patients were reclassified from ASPECTS ≤ 5 to ASPECTS ≥ 6 with DLIR-M, making them eligible for endovascular therapy. Using FBP instead of DLIR-M, these patients might have been excluded from thrombectomy. Additionally, DLIR-M showed the highest agreement between the consensus expert ground truth and the automated ASPECTS.

Our study has several limitations. The evaluation was performed using reconstruction algorithms from a single CT manufacturer, which may limit the generalizability of the findings to other systems. Secondly, in this study, we did not analyze the accuracy of the segmentation procedure as part of the automated ASPECT, as well as the influence of possible interferences such as leukarariosis. This initial segmentation step for automated ASPECT would benefit from further studies to ensure optimization and further refinement. While FBP was employed as the reference standard, it is not a definitive method for ASPECTS due to the lack of a universally accepted gold standard. Moreover, we acknowledge the limited sample size of our study, larger multicenter studies are essential to confirm the robustness of the conclusions. Furthermore, dose reduction was not incorporated into the CT acquisition protocol, although deep learning-based reconstruction and iterative reconstruction techniques have previously shown potential for dose reductions in head CT [35, 36]. This was due to the retrospective character of this study and ethical considerations, since a correct ASPECTS has therapeutic consequences and may outweigh radiation protection in the acute emergency setting. Additionally, due to the retrospective nature of this study, simultaneous diffusion-weighted MRI, which could likely provide the most accurate ground truth in cases of acute stroke, was not feasible in the present study. We attempted to compensate for this by establishing an expert-based ground truth for comparing automated and reader-based ASPECT, with access to clinical information, baseline CT, CT angiography, and CT perfusion imaging, as well as follow-up imaging. However, as the comparison was limited to total ASPECTS between the consensus assessment and automated evaluation of different reconstruction techniques, discrepancies within individual ASPECTS regions were not analyzed.

In conclusion, different CT reconstruction techniques resulted in only a minor underestimation of automated ASPECTS compared to FBP. Especially the insula and the cortical M3 ASPECTS seemed more susceptible to algorithmic variability. However, more patients were overestimated from ASPECTS ≤ 5 to ASPECTS ≥ 6, making them available for endovascular therapy, which was most pronounced for DLIR-M. DLIR-M also exhibited the highest agreement with consensus expert ground truth for automated ASPECTS. Careful selection of reconstruction parameters, as well as further optimization and standardization of these techniques as well as automated segmentation, is therefore essential for broader application in stroke imaging to minimize discrepancies and improve consistent and reliable patient management. Furthermore, this work might serve as an important foundation for future AI-based research by pointing out that different reconstruction techniques must be taken into account when applying AI in neuroradiological research.

## Supplementary Information

Below is the link to the electronic supplementary material.

Supplementary Material 1

## Data Availability

No datasets were generated or analysed during the current study.
