# Supplementary material for: Influence of deep learning image reconstruction and adaptive statistical iterative reconstruction-V on automated Alberta stroke program early CT score- evaluation
Source: Neuroradiology. 2026 Feb 18;68(4):953–63. doi: 10.1007/s00234-026-03938-5 (PMC13139252; doi:10.1007/s00234-026-03938-5)
Supplement: Supplementary file 1 — Supplementary Material 1 [file 234_2026_3938_MOESM1_ESM.docx]

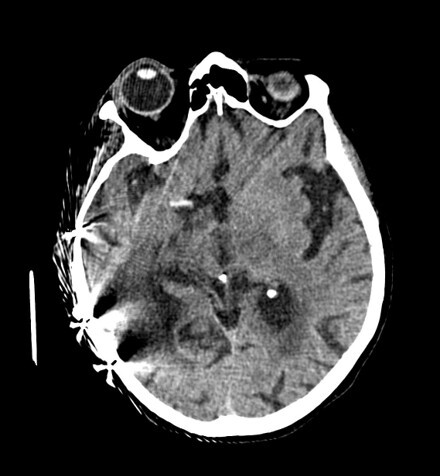


**Suppl. Fig. 1** Axial non-contrast CT scan with hyperdense MCA sign on the right side of a 91-year-old patient presenting with left-sided hemiplegia, ipsilateral horizontal gaze palsy (to the left) with déviation conjugée and anarthria. The patient was excluded from the study due to severe artifacts caused by a cochlear implant in the right temporal region.
